# The outcomes and prognostic factors of patients who underwent reoperation for persistent/recurrent papillary thyroid carcinoma

**DOI:** 10.1186/s12893-022-01819-1

**Published:** 2022-11-02

**Authors:** Wenyu Sun, Lu Di, Lili Chen, Duanshu Li, Yi Wu, Jun Xiang, Shichong Zhou, Tuanqi Sun

**Affiliations:** 1 Department of Head and Neck Surgery, Fudan University Shanghai Cancer Center, No. 270, Dong’an Road, Shanghai, 200032 China; 2 Department of Oncology, Shanghai Medical College of Fudan University, Shanghai, China; 3 Department of Internal Medicine, Wusong Hospital, Zhongshan Hospital, Fudan University, Shanghai, China; 4 Department of Ultrasound, Fudan University Shanghai Cancer Center, No. 270, Dong’an Road, Shanghai, 200032 China

**Keywords:** Reoperation, Radioiodine therapy, Papillary thyroid carcinoma, Recurrence

## Abstract

**Background:**

While the most suitable approach for treating persistent/recurrent papillary thyroid carcinoma (PTC) remains controversial, reoperation may be considered an effective method. The efficacy of reoperation in patients with locoregional persistent/recurrent PTC, especially those with unsatisfactory radioactive iodine (RAI) ablation results, is still uncertain. This study aimed to clarify the clinical management strategies for locoregional persistent/recurrent PTC and to explore factors that may affect long-term patient outcomes after reoperation.

**Methods:**

In total, 124 patients who initially underwent thyroidectomy and variable extents of RAI therapy and finally received reoperation for locoregionally persistent/recurrent PTC were included. The parameters associated with recurrence-free survival (RFS) were analysed using a Cox proportional hazards model.

**Results:**

Overall, 124 patients presented with structural disease after initial therapy and underwent secondary surgical resection, of whom 32 patients developed further structural disease during follow-up after reoperation. At the time of reoperation, metastatic lymph nodes with extranodal extension (*P* = 0.023) and high unstimulated thyroglobulin (unstim-Tg) levels after reoperation (post-reop) (*P* = 0.001) were independent prognostic factors for RFS. Neither RAI avidity nor the frequency and dose of RAI therapies before reoperation affected RFS.

**Conclusions:**

Reoperation is an ideal clinical treatment strategy for structural locoregional persistent/recurrent PTC, and repeated empirical RAI therapies performed prior to reoperation may not contribute to the long-term outcomes of persistent/recurrent PTC patients. Metastatic lymph nodes with extranodal extension and post-reop unstim-Tg > 10.1 ng/mL may predict a poor prognosis.

**Supplementary Information:**

The online version contains supplementary material available at 10.1186/s12893-022-01819-1.

## Introduction

Papillary thyroid carcinoma (PTC) is the most common well-differentiated thyroid cancer derived from thyroid follicular cells. Although favourable prognoses are achieved in most patients who undergo standardized surgical treatment, postoperative suppressive therapy and radioiodine (RAI) therapy if necessary, locoregional persistent/recurrent PTC is still frequently observed, especially in those classified as high risk by the risk stratification system [1]. Due to the unclear prognostic value of this approach for locoregional persistent/recurrent PTC [2, 3], there is a lack of consensus regarding the clinical management of structural locoregional recurrence [4].

In the most recent American Thyroid Association (ATA) guidelines, an authoritative guidance for clinical professionals, the management of metastatic disease was introduced as surgical excision of locoregional diseases, RAI therapy for RAI-responsive disease, and other nonsurgical approaches [1]. Several studies have illustrated that reoperation is considered an optimal and effective approach to cervical structural persistent/recurrent disease, although they lack long-term follow-up data [5–9]. However, given the complexity and high risks of repeat surgery, especially when involving compartments operated upon previously and disrupted anatomic planes from the initial surgery [10], many clinical practitioners prefer nonsurgical and other more conservative approaches to treat locoregional recurrence [11, 12].

Maxon et al. demonstrated that RAI is a successful strategy for treating metastatic cervical lymph node disease [13]. Furthermore, it has been shown that if persistent/recurrent cervical lymph node disease presents with RAI-avid characteristics, RAI therapy would be an ideal option [14]. Younger patients presenting with RAI-avid low-volume metastases may benefit from RAI therapy, despite the need for repeated treatments [15]. Repeated RAI therapy may allow some patients to avoid a secondary surgical intervention; however, we have observed in our clinical practice that many patients with persistent/recurrent disease eventually have to opt for reoperation, even after various extents of RAI therapy, ranging from a single ablation to repeated empirical treatments.

The main purpose of this study was to extend our understanding of the effectiveness of RAI therapy performed before reoperation for cervical persistent/recurrent PTC. Particularly, we attempted to identify the avidity of RAI therapy and distinguish patients with iodine-refractory locoregional persistent/recurrent PTC from those presenting with RAI-avid disease based on the post-therapy whole-body scan (WBS). Herein, we further discuss whether RAI avidity and additional RAI therapy could influence patient outcomes after reoperation and investigate other parameters that could predict secondary locoregional structural recurrence after reoperation to clarify the clinical management strategies for patients with locoregional persistent/recurrent PTC.

## Patients and methods

### Patients

**Fig. 1:**
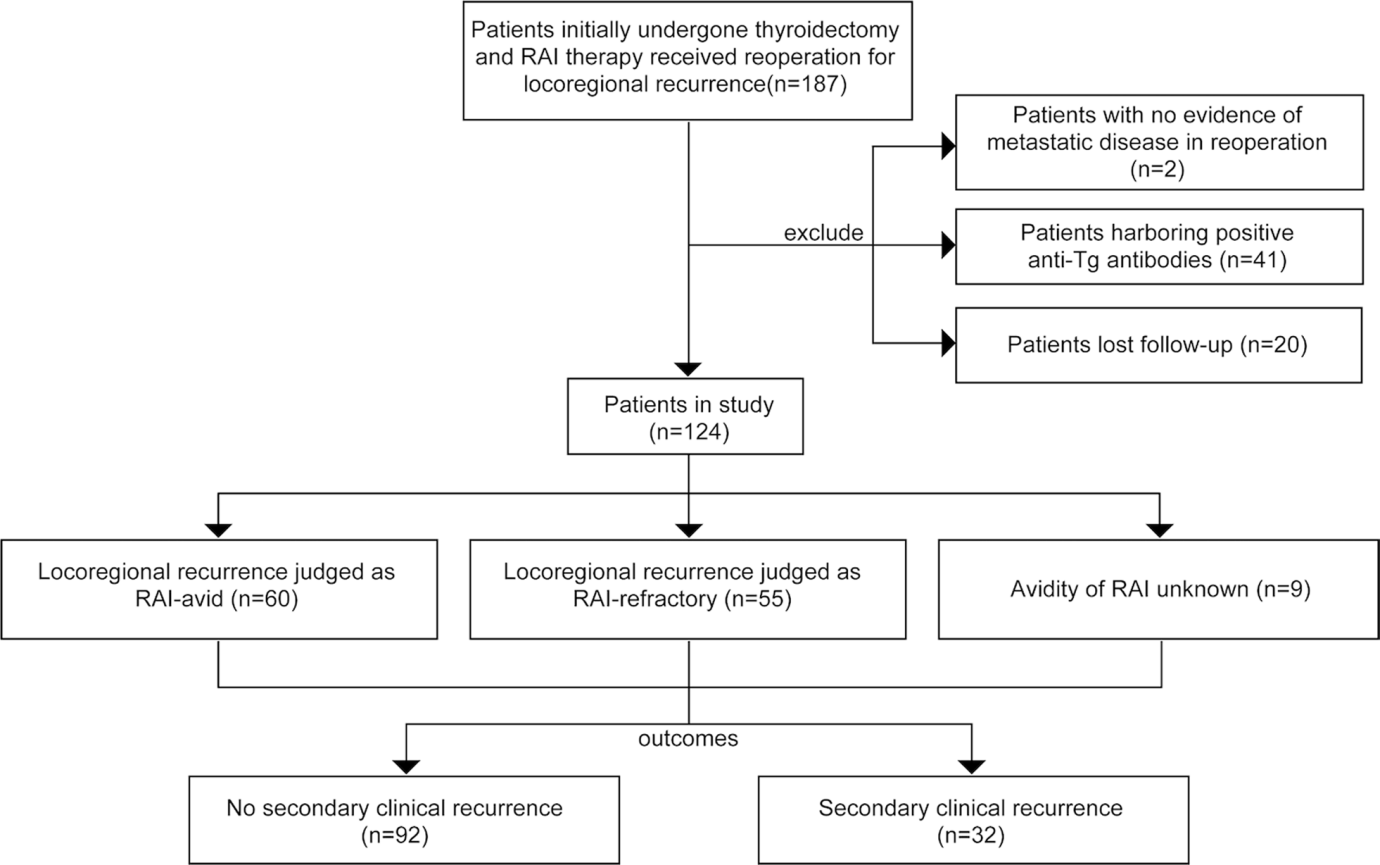
Description of the study cohort

A total of 187 patients with PTC underwent reoperation for recurrent/persistent locoregional structural disease at Fudan University Shanghai Cancer Center (FUSCC) from 2007 to 2017. All patients were diagnosed with persistent or newly identified locoregional metastases after initial surgery (thyroidectomy with or without lateral neck dissection) and RAI therapy; the diagnosis was highly dependent on the availability of structural/functional imaging and included both surveillance WBS iodine scans and ultrasound (US) that detected highly suspicious lymph nodes with/without US-guided fine needle aspiration (FNA) cytology results and suspicious structural disease observed by other imaging methods. All patients received various empirical radioiodine therapy regimens before reoperation, and no evidence of distant metastasis was observed in these patients. Of the total population, two patients with no evidence of metastatic disease in the surgical specimen and 20 patients lost to follow-up were excluded. In total, 41 patients harbouring positive anti-thyroglobulin antibody (TgAb) expression were also excluded (Fig. 1).

The parameters collected in the study are listed in Table 1. This study was approved by ethics board of Fudan University Shanghai Cancer Center (No.:050432-4-1911D) and and informed consent forms were signed by all the patients.

**Table 1:** Clinical characteristics of 124 patients included in the study

Variables	Median(range)/number(ratio)
Female	80/ (64.5%)
Age at reoperation	42 (13–73)
Time span between initial surgery and reoperation (months)	16 (5–38)
Pre-reop unstim-Tg (ng/mL)^a^	11.12 (0.1–1000)
Post-reop unstim-Tg (ng/mL)^b^	2.04 (0.1–600)
Tg different between pre-/post-reoperation (ng/mL)^c^	5.03 (− 499.82–999.61)
Frequency of pre-reop RAI therapy	2 (1–8)
Dose of pre-reop RAI therapy (mCi)	250 (50–750)
RAI-avidity character	
Avid	60 (48.4%)
Refractory	55 (44.4%)
Unknown	9 (7.2%)
Number of pathologically proven metastatic lymph node at reoperation	4 (1–19)
Largest dimision of positive lymph node (cm)	1.9 (0.4–5)
Metastatic lymph node with extranodal extension	39 (31.4%)
Metastatic lymph node with squamous dedifferentiation	6 (4.8%)

^a^Pre-reop unstim-Tg were performed at the time just before reoperation

^b^Post-reop unstim-Tg were performed 1–3 months after reoperation

^c^The Tg different was calculated by subtracting post-reop unstim-Tg from pre-reop unstim-Tg

*Pre-reop* pre-reoperative, *Post-reop* post-reoperative, *unstim-Tg* unstimulated thyroglobulin, *RAI* radioiodine, *cm* centimeter

### Extent of reoperation

Reoperation for persistent/recurrent disease included central and/or lateral neck dissection (CND/LND, ipsilateral or bilateral). The extent of reoperation was totally based on the anatomic area of disease that appeared suspicious on imaging or clinically evident nodal disease, regardless of whether the compartments in question were dissected in previous surgery. All complementary CNDs were repeated but were limited by the previous surgery, while complementary LND may be radical if the lateral area had not been dissected before (lateral modified radical neck dissection involved levels IIa–Vb). If the complementary LND was performed on a previously dissected part related to the scarred and fibrotic tissue, the surgery may be limited due to the scarring/distortion of the anatomy from the initial dissection and RAI therapy (the level of structural disease involved along with the most adjacent compartment but may not extend to the original anatomical boundary of these levels). Some reoperations also involved level VII subcompartments and resection of local persistent/recurrent disease with aerodigestive invasion. Neither node plucking nor berry picking techniques were involved in our reoperation.

The extent of the neck dissection area in the initial surgery and reoperation, along with the clinicopathologic features of the primary tumour, are listed in Additional file 1: Table S1.

### Follow-up methods and the definition of secondary structural recurrence after reoperation

Follow-up modalities included physical examination, US, unstim-Tg, neck and chest computed tomography (CT) scan, and ^131^I planer WBS with or without single-photon emission computed tomography (SPECT). Some patients also underwent fluorodeoxyglucose positron emission tomography (FDG-PET).

Secondary locoregional structural recurrence was defined as the locoregional reappearance of pathologically proven malignant tissue in the second or third reoperation procedure or FNA biopsy or the appearance of distant metastases in the mediastinum, bone, lung, or brain as evidenced by ^131^I WBS or other imaging methods, such as ^18^FDG PET-CT, during the follow-up period after reoperation.

To estimate the risk of recurrence through long-term follow-up after initial treatment, Tuttle et al. established the “Response to Therapy” stratification, an approach widely employed in clinical studies [16, 17]. This stratification was adopted in this study to categorize the long-term outcomes of the patients presenting with secondary structural recurrence into 4 dimensions: (1) excellent response (ER), including negative imaging findings and unstim-Tg < 0.2 ng/mL, (2) biochemical incomplete response (BIR), including unstim-Tg > 1 ng/mL with negative imaging findings, (3) indeterminate response (IR), including nonspecific findings on imaging studies and unstim-Tg between 0.2 and 1 ng/mL, and (4) structural incomplete response (SIR), including structural evidence of disease regardless of Tg level.

### Definition of RAI-refractory and RAI-avid locoregional recurrence

To discern the recurrent metastatic nodules as either radioiodine avid (RAI-A) or refractory (RAI-R) more precisely, nine patients who received remnant ablation as the primary goal of RAI administration after thyroidectomy were further excluded, as the lymph node metastases of these patients were found during late follow-up, far from the initial RAI treatment; therefore, RAI avidity was not determinable. The remaining 115 patients had cervical recurrences that were classified as either RAI-A or RAI-R according to postablation and posttherapy WBS results or routine diagnostic WBS with or without SPECT at follow-up. A minority of patients who had ^18^FDG uptake on PET-CT were classified as RAI-R.

### Statistics

For clinical characteristics, continuous variables are reported as the medians and ranges, and categorical variables are reported as frequencies and percentages. Cut-off values for continuous variables such as unstim-Tg level, age, the time between initial surgery and reoperation, and the frequency and dose of RAI therapy were determined by the X-tile program (Yale University School of Medicine, New Haven, CT, USA, RRID:SCR_005602). Recurrence-free survival (RFS) curves were drawn and analysed using the Kaplan–Meier method, and differences in RFS were calculated using log-rank tests. Risk factors associated with RFS were analysed using a Cox proportional hazards model. Analyses were performed with SPSS version 22.0 (IBM Corporation, Chicago, IL, USA, RRID:SCR_002865). A two-sided *P* value < 0.05 was considered statistically significant.

## Results

### Clinical characteristics of patients undergoing reoperation

As shown in Fig. 1, 124 patients were included in our study (median age at reoperation: 42 years, range: 13–73 years). For RAI therapy, the median cycles and dosage of preoperative RAI therapy were 2 cycles [1–8 times] and 250 mCi (80–750 mCi), respectively. The patients were classified into three groups based on the RAI avidity of their locoregional recurrences: the RAI-A group comprised 60 patients, the RAI-R group comprised 55 patients, and the remaining 9 patients were classified as having unknown RAI avidity. A median of 4 lymph nodes [1–19] were pathologically proven to be positive at reoperation. Extranodal extension was present in the metastatic lymph nodes of 39 patients, whereas squamous dedifferentiated characteristics were present in the lymph nodes of 6 patients. The median follow-up duration after reoperation was 36 months (1–120 months). Of the 124 patients, 32 patients were diagnosed with secondary structural recurrence (Table 1).

### Univariate and multivariable analysis of secondary structural recurrence

**Fig. 2:**
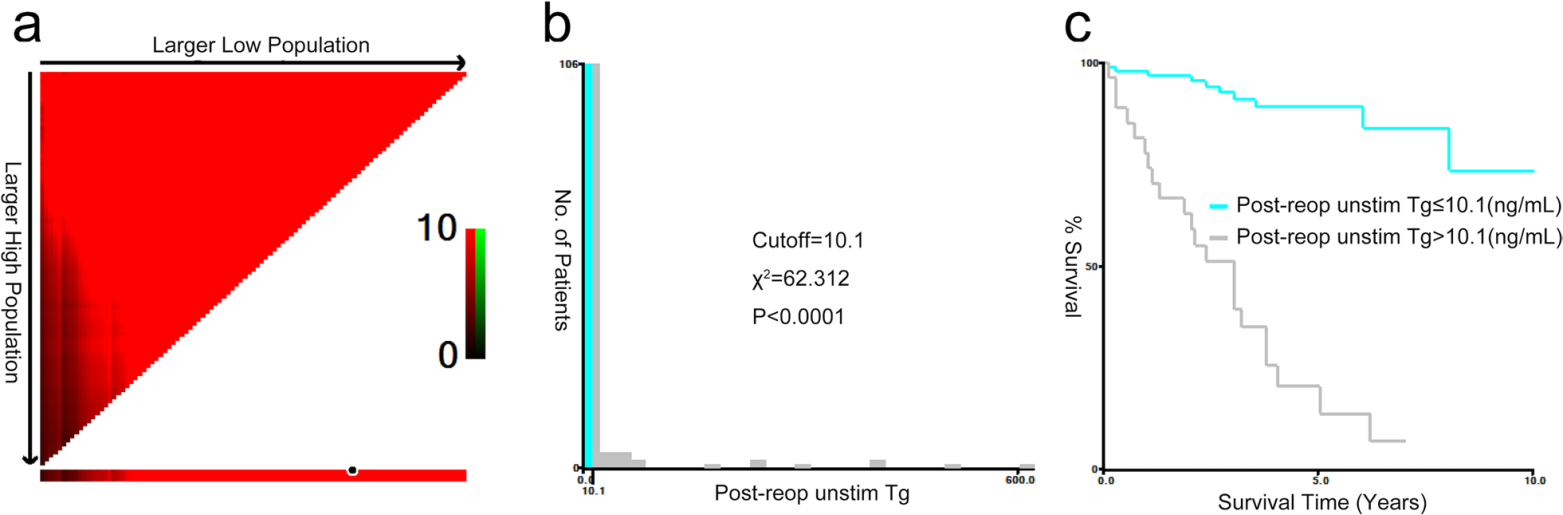
Left panel (**a**) showed the X-tile plots of training sets. The optimal cut-points highlighted by the black circle in the left panel were shown on histograms of the entire cohort displayed in the middle panel (**b**) and Kaplan–Meier estimates of two subgroups, defined by having post-reoperative unstim-Tg levels either higher or lower than 10.1 ng/mL, showed a significant difference in RFS (log-rank value = 62.312, *P* < 0.001, Fig. 2), dividing the cohort into high- and low-risk subgroups displayed in the right panel (**c**)

Univariable analysis showed that age > 55 years, unstimulated thyroglobulin (unstim-Tg) levels after reoperation (post-reop) > 10.1 ng/mL, time between first and secondary surgery > 23 months, RAI therapy cycles > 3 times, RAI dose > 440 mCi, and metastatic lymph nodes with extranodal extension were associated with secondary structural recurrence in patients after reoperation. Multivariate analysis using a Cox proportional hazards model showed that only metastatic lymph nodes with extranodal extension (HR = 3.129; 95% CI = 1.167–8.392, *P* = 0.023) and post-reop unstim-Tg > 10.1 ng/mL (HR = 4.597; 95% CI = 1.881–11.233, *P* = 0.001) were independent prognostic factors of RFS (Table 2, Fig. 2).

**Table 2:** Univariable and multivariable analysis for recurrence free survival (RFS) in 124 patients

Variables	Univariable analysis		Multivariable analysis	
	*P* value	HR (95%CI)	*P* value	HR (95%CI)
Gender				
Male	0.954	0.979(0.478–2.007)		
Female		1		
Age at reoperation*				
> 55	0.003	3.103 (1.487–6.477)	0.297	1.528 (0.689–3.392)
≤ 55		1		1
Post-reop unstim-Tg (ng/mL)*				
> 10.1	0	11.135 (5.14–24.122)	**0.001**	4.597 (1.881–11.233)
≤ 10.1		1		1
Time span between initial surgery and reoperation (months)*				
> 23	0	6.003 (2.93–12.298)	0.213	1.797 (0.714–4.522)
≤ 23		1		1
Frequency of pre-reop RAI therapy*				
> 3 times	0.001	3.371 (1.678–6.771)	0.376	0.472 (0.09–2.484)
≤ 3 times		1		
Dose of pre-reop RAI therapy (mCi)*				
> 440	0	5.305 (2.641–10.656)	0.204	2.742 (0.579–12.983)
≤ 440		1		1
RAI-avidity character				
	0.58			
RAI-Avid	0.297	1.471 (0.712–3.041)		
Unknown	0.737	1.294 (0.289–5.802)		
RAI-refractory		1		
Metastatic lymph node with extranodal extension				
Yes	0	9.096 (4.049–20.435)	**0.023**	3.129 (1.167–8.392)
No		1		1

All *P* values < 0.05 are shown in bold

*The cutoff value of age, unstim-Tg level, time span between initial surgery and reoperation, frequency and dose of RAI therapies were determined by X-tile program

*HR* hazard ratio, *CI* confidence interval, *RAI* radioiodine

### Long-term outcomes of patients with secondary structural recurrence

Of the 32 patients with secondary structural recurrence, proven either pathologically or by definitive imaging methods, 23 patients had secondary recurrence in their cervical lymph nodes and underwent a second or third reoperation. The median follow-up time from reoperation to secondary recurrence was 24 months (1–96 months). Of these patients, six (26%) were finally classified as achieving an ER, five (21%) as BIR, three (13%) as SIR, and three (13%) as IR; four (17%) eventually developed metastases to their lungs and bones, whereas two developed para-tracheal recurrence, which evolved into aerodigestive invasion unamenable to surgery, and these patients were finally enrolled in kinase inhibitor therapy (sorafenib clinical trial). The other nine patients had distant metastases as secondary structural recurrence, and the median follow-up time was 32 months (11–74 months). Among these patients, two of the metastases were in the mediastinum, three were in the lung, one was in the bone, and one was solely in the brain, whereas the remaining two patients simultaneously developed multiple of the abovementioned metastases.

### Squamous dedifferentiated components in recurrent cervical lymph nodes

It was observed that pathologically squamous dedifferentiated components in recurrent cervical lymph nodes were associated with well-differentiated metastatic PTC in six patients (4.8%) who underwent repeated empirical RAI therapies before reoperation. All these recurrent metastatic nodes showed RAI-avid characteristics initially but eventually were classified into the RAI-R group according to the post-therapy WBS. In long-term follow-up, two of the six patients had no secondary recurrences, two patients developed distant metastasis in the lungs and bones, and the remaining two patients developed secondary recurrences in the cervical lymph nodes and underwent a second reoperation, although they eventually developed either metastases in the clavicle or tracheal invasion.

## Discussion

Locoregional persistent/recurrent disease was most common after initial thyroidectomy and active radioiodine therapy in PTC patients. Clinical management entails comprehensive clinical management facilitated by surgeons, endocrinologists, and nuclear medicine specialists [18]. However, the emotional factors of the patients and their families should be equally emphasized in the clinical decision-making process [19]. There are many variables that should be considered, and the decision of how best to manage individual patients with persistent/recurrent PTC is always complex. The most recent ATA guidelines proposed that these management strategies include reoperation, active surveillance, 131I therapy for RAI-responsive disease, external beam radiation therapy and some other nonsurgical modalities [1, 4]. The guidelines arranged these approaches in a hierarchy, demanding surgical excision prior to other management, and these recommendations were based on a series of clinical studies concerning the effectiveness of secondary surgery. Nevertheless, only 5 of these studies provided the number and ratio of patients who have received RAI after primary surgery, depicting these treatment procedures as failed RAI therapy or just RAI ablation [5, 6, 9, 20, 21]. Several studies have judged the role of RAI therapy after reoperation [22, 23]. Overall, these studies seldom offer clear suggestions of radioiodine prior to reoperation, illustrate the clinical response to RAI therapy and radioiodine avidity of these persistent recurrences and whether RAI could influence the outcome after reoperation.

RAI avidity is a critical factor that may impact the treatment decision, in that RAI-avid lymph node metastases may indicate a strong probability of clinical response to RAI therapy. As such, the literature treats RAI avidity as a negative indicator for secondary surgical intervention. Practically, however, it is not a simple procedure to distinguish RAI-A disease from RAI-R disease. Consistent with that described in the ongoing classification of RAI-R PTC [24], this study also found that in an individual patient, the response of distinct lymph node metastasis to iodine can vary from RAI-A to RAI-R, making it hard to distinguish in the planar WBS and even in the cross-sectional SPECT image. Some patients who initially present with RAI avidity may gradually lose this indicator over repeated therapies. In contrast, some recurrences remain RAI-A for the duration of treatment, with Tg levels decreasing slightly and structural components remaining stable rather than progressing as per the Response Evaluation Criteria in Solid Tumors (RECIST). Because of these differences in presentation, the decision to assess RAI avidity can be difficult or even controversial; patients may endure ineffective empirical RAI therapies before clinicians discover the RAI-R characteristics of recurrent nodes initially assumed to be RAI avid. In this study, neither a more cycles and higher doses of RAI therapy nor the response dynamics of the metastatic nodes to RAI therapy affected the prognosis after secondary surgical intervention; in fact, delayed reoperation may lead to a poor prognosis. This serves as a reminder that there is no need to perform repeated empirical RAI therapies to discern RAI avidity, and since the criteria for RAI avidity have not yet been established, RAI therapies should be used more judiciously.

**Fig. 3:**
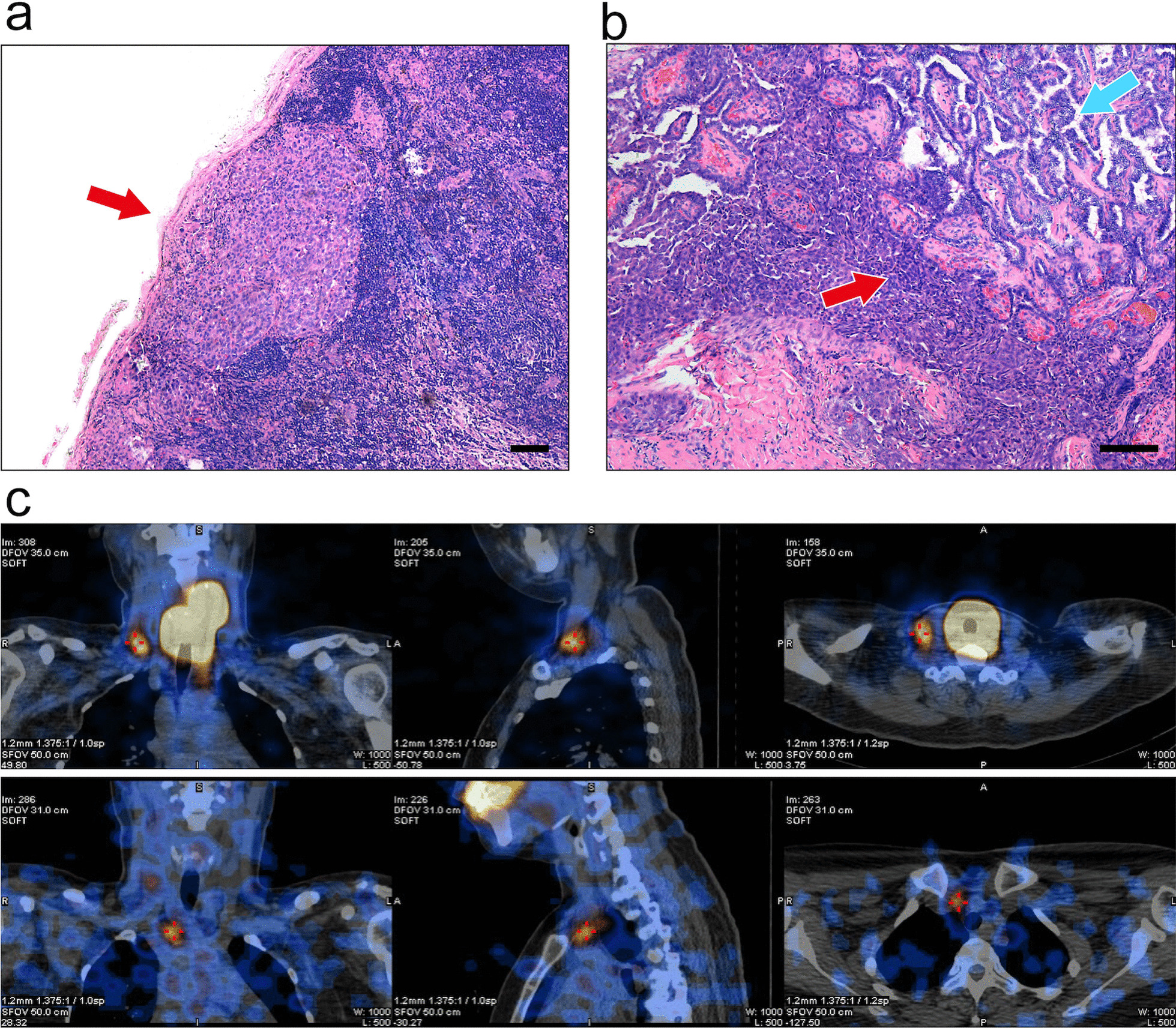
The hematoxylin–eosin (HE) staining of one recurrent metastatic lymph node with squamous dedifferentiation dissected in reoperation and the pre-reoperation I^131^ SPECT image. The squamous dedifferentiation component locates at the edge of this metastatic lymph node (red arrow) (**a**) The squamous dedifferentiation component (red arrow) is completely detached and separate from the classic PTC metastasis component (blue arrow) in the metastasis lymph node (**b**) The metastatic lymph node (red cross) persistently present RAI-avid character in the post-therapy. ^131^I WBS with SPECT after 2 courses of RAI therapy (150 + 200 mCi), the uptake in central neck around the trachea is the local remnant in thyroid bed (**c**)

Repeated RAI therapy has been recommended by the ATA guidelines to treat persistent nonresectable disease that is visualized and reduced after RAI therapy until eliminated [1]. However, Sabra et al. demonstrated that there is little benefit to repeated empirical RAI for distant metastasis, even for RAI-avid metastases on post-therapy WBS [25]. In our study, the histopathological characteristics of locoregionally persistent/recurrent PTC were analysed, which may provide a unique perspective that can help elucidate why these metastatic lesions persist and require surgical intervention, despite showing RAI avidity on post-therapy WBS. We found squamous dedifferentiated components in the recurrent cervical lymph node specimens. This was associated with a well-differentiated, metastatic PTC component after exposure to repeated empirical RAI therapy before reoperation. The squamous dedifferentiated components were entirely detached and separated from the classic metastatic PTC components in the lymph nodes and may structurally persist while the RAI-avid classic PTC component is ablated after repeated empirical RAI therapy, thereafter revealing the RAI-R characteristics of this component (Fig. 3). Ito et al. and Beninato et al. described the aggressive behaviour and poor long-term outcomes associated with squamous dedifferentiation in primary PTC tumours and lymph node metastases [26, 27]. In our study, histology after reoperation confirmed the invasive biological characteristics of the squamous dedifferentiated metastatic lymph nodes. All these patients presented with classic PTC in their primary tumours and had no evidence of squamous cell carcinoma in other sites of the body; however, we were unable to assess and evaluate the relationship between changes in squamous dedifferentiation and repeated RAI therapies due to the small sample size.

Other parameters that affect the management of persistent/recurrent disease include the size of the lymph nodes. The size threshold for surgical intervention to treat lymph nodes is 8 mm (mm) in the central neck compartment and 10 mm in the lateral neck compartment; these cut-off dimensions may be the least amenable to US-guided FNA and be anatomically feasible during reoperation. The literature increasingly suggests that the recommended threshold of 8 mm be raised to 15 mm in central nodes because a size > 15 mm can be an independent risk factor for BIR and is associated with a higher risk of local invasion and surgical morbidity, while a size of 10–15 mm is not [28]. In this study, no correlation was found between lymph node size and invasive biological characteristics; the size of the lymph nodes did not have any prognostic value for secondary recurrence. Furthermore, the smallest dimensions of a metastatic lymph node in one of our patients were 6 × 5 × 5 mm, indicating that the threshold may not be a uniform standard in experienced hands. We used anatomic marks on skin and adopted charcoal tattooing for the lymph nodes under US guidance before reoperation to help anatomically localize recurrent/persistent disease, and surgical methods may be feasible for structural disease with smaller dimensions in our study [29, 30].

To our knowledge, this is the first study on the utility of RAI therapy before reoperation and the first to provide clinical and pathological evidence that neither extended empirical RAI therapies before reoperation nor RAI avidity had effects on the long-term outcomes of patients with locoregional recurrence of PTC. Only metastatic lymph nodes with extranodal extension or a post-reop unstim-Tg > 10.1 ng/mL were independent prognostic factors for RFS. The limitation of our study is that we didn’t present results for patients who received RAI therapy only for their recurrent/persistent disease and got an excellent response or a tolerable response (BIR/IR) in long-term follow-up, thus we may neglects some structural cases that may be truly RAI-Avid and can be totally ablated solely by RAI. However, given the clinical complexity of distinguishing RAI-R from RAI-A, unless there is a clear radioiodine response to RAI (significant Tg decrease/structural reduction), more repeated empirical radioiodine therapy should not be used to identify avidity to RAI and timely surgical intervention should be taken into consideration, which is an effective clinical management strategy for these patients. Further investigations on molecular markers for RAI-A and RAI-R lymph node metastases may assist in understanding the genetic mechanisms involved in the loss of RAI avidity and the dedifferentiation process for PTC cells, which may play a critical role in the therapeutic decision-making process for persistent/recurrent PTC.

## Supplementary Information

**Additional file 1: Table S1.** The clinicopathologic feature of primary tumor and the extent of neckdissection area in initial surgery/reoperation.

## Data Availability

The datasets used and analysed during the current study are available from the corresponding author on reasonable request.

## Abbreviations

ATA: American thyroid association
BIR: Biochemical incomplete response
CND: Central neck dissection
ER: Excellent response
FDG-PET: Fluorodeoxyglucose positron emission tomography
FNA: Fine needle aspirate
FUSCC: Fudan University Shanghai Cancer Center
IR: Indeterminate response
LND: Lateral neck dissection
post-reop: Post-reoperative
PTC: Papillary thyroid carcinoma
RAI: Radioiodine
RAI-A: Radioiodine-avid
RAI-R: Radioiodine-refractory
RECIST: Response evaluation criteria in solid tumors
RFS: Recurrence free survival
SIR: Structural incomplete response
SPECT: Single-photon emission computed tomography
Tg: Thyroglobulin
unstim: Unstimulated
US: Ultrasound
WBS: Whole body scan
