# Supplementary material for: The outcomes and prognostic factors of patients who underwent reoperation for persistent/recurrent papillary thyroid carcinoma
Source: BMC Surg. 2022 Nov 2;22:374. doi: 10.1186/s12893-022-01819-1 (PMC9632153; doi:10.1186/s12893-022-01819-1)
Supplement: Supplementary file 1 — Additional file 1: Table S1. The clinicopathologic feature of primary tumor and the extent of neckdissection area in initial surgery/reoperation. [file 12893_2022_1819_MOESM1_ESM.docx]

Table S1. The clinicopathologic feature of primary tumor and the extent of neck dissection area in initial surgery/reoperation

| The clinicopathologic feature in initial surgery | Number（ratio） |
| --- | --- |
| T stage |  |
| pT1 | 64(52%) |
| pT2 | 36(29%) |
| pT3 | 16(13%) |
| pT4 | 8(6%) |
| N stage |  |
| pN0 | 11(9%) |
| pN1a | 56(45%) |
| pN1b | 57(46%) |
| AJCC staging |  |
| I/II | 103(83%) |
| III/IV | 21(27%) |
| Neck dissection in initial surgery |  |
| Ipsilateral CND | 43(35%) |
| Bilateral CND | 24(19%) |
| Ipsilateral CND+LND | 41(33%) |
| Bilateral CND+LND | 16(13%) |
| Extent of Reoperation |  |
| complemental-CND solely | 34(27%) |
| radical-LND (with/without complemental-CND) | 48(39%) |
| complemental LND (with/without complemental-CND) | 42(34%) |

CND: central neck dissection LND: lateral neck dissection
